# Anti-Leukemic Properties of Histamine in Monocytic Leukemia: The Role of NOX2

**DOI:** 10.3389/fonc.2018.00218

**Published:** 2018-06-18

**Authors:** Roberta Kiffin, Hanna Grauers Wiktorin, Malin S. Nilsson, Johan Aurelius, Ebru Aydin, Brianna Lenox, Jonas A. Nilsson, Anders Ståhlberg, Fredrik B. Thorén, Kristoffer Hellstrand, Anna Martner

**Affiliations:** Sahlgrenska Cancer Center, University of Gothenburg, Gothenburg, Sweden

**Keywords:** histamine, NAPDH oxidase, NOX2, acute myeloid leukemia, acute monocytic leukemia, acute myelomonocytic leukemia

## Abstract

In patients with acute myeloid leukemia (AML), treatment with histamine dihydrochloride (HDC) and low-dose IL-2 (HDC/IL-2) in the post-chemotherapy phase has been shown to reduce the incidence of leukemic relapse. The clinical benefit of HDC/IL-2 is pronounced in monocytic forms of AML, where the leukemic cells express histamine type 2 receptors (H_2_R) and the NAPDH oxidase-2 (NOX2). HDC ligates to H_2_Rs to inhibit NOX2-derived formation of reactive oxygen species, but details regarding the anti-leukemic actions of HDC remain to be elucidated. Here, we report that human NOX2^+^ myelomonocytic/monocytic AML cell lines showed increased expression of maturation markers along with reduced leukemic cell proliferation after exposure to HDC *in vitro*. These effects of HDC were absent in corresponding leukemic cells genetically depleted of NOX2 (*NOX2*^−/−^). We also observed that exposure to HDC altered the expression of genes involved in differentiation and cell cycle progression in AML cells and that these effects required the presence of NOX2. HDC promoted the differentiation also of primary monocytic, but not non-monocytic, AML cells *in vitro*. In a xenograft model, immunodeficient NOG mice were inoculated with wild-type or *NOX2*^−/−^ human monocytic AML cells and treated with HDC *in vivo*. The administration of HDC reduced the *in vivo* expansion of *NOX2*^+/+^, but not of *NOX2*^−/−^ human monocytic AML cells. We propose that NOX2 may be a conceivable target in the treatment of monocytic AML.

## Introduction

Acute myeloid leukemia (AML) is characterized by unrestrained growth of myeloid cells in bone marrow (BM) and other organs. The initial treatment comprises induction and consolidation chemotherapy aimed at inducing and sustaining the disappearance of leukemic cells (complete remission, CR) (1). However, most AML patients will experience life-threatening relapses in the post-chemotherapy phase, likely as the result of expansion of residual leukemic cells, and few treatments are available to prevent relapse (2, 3). Results from a phase III trial showed that post-remission treatment with the histamine derivative histamine dihydrochloride (HDC) in conjunction with low-dose IL-2 (HDC/IL-2) significantly improved leukemia-free survival (4) and meta-analyses supported that the HDC component was critical for the clinical efficacy of this regimen (5, 6). In addition, *post hoc* analyses of phase III trial results suggested that HDC/IL-2 may be preferentially or specifically efficacious in patients where the leukemic clone is dominated by monocytic cells, i.e., myelomonocytic (FAB class M4) or monocytic (M5) AML (7). Details regarding the mechanisms of anti-leukemic activity of HDC/IL-2 have only partially been explored, in particular regarding the contribution by HDC (8, 9).

The NAPDH oxidase-2 (NOX2) enzyme is the major source of reactive oxygen species (ROS) that constitute an essential feature of the innate antimicrobial defense mediated by myeloid cells (8, 10, 11). Additionally, ROS have been implicated in myeloid cell differentiation as high levels of intracellular ROS hinder proper differentiation of myeloid cells into dendritic cells (DC) and macrophages (12). Furthermore, NOX2-derived ROS were recently found to facilitate mitochondrial transfer from BM stromal cells to AML cells, which may enhance metabolism and survival of AML cells (13). Extracellular release of ROS from myeloid cells may also trigger dysfunction and apoptosis of adjacent cells, including elements of lymphocyte-mediated immunity (14). HDC reduces the NOX2-dependent ROS formation *via* histamine type 2 receptors (H_2_R) expressed by myeloid cells (14, 15). By this mechanism, HDC safeguards natural killer cells and cytotoxic T cells from apoptosis inflicted by neighboring ROS-producing myeloid cells, thus facilitating immune-mediated elimination of malignant cells (14, 16–18).

The ability of HDC to protect immune cells with tumor-killing capacity has been proposed to contribute to the clinical benefit of HDC-based therapy in AML (8, 19, 20). Anti-leukemic properties of HDC may alternatively or additionally relate to its pro-differentiating effects on myeloid cells. Yang et al. thus reported that genetic disruption of endogenous histamine formation in mice, with ensuing depletion of histamine from tissues, resulted in the accumulation of immature CD11b^+^Gr1^+^ myeloid cells in blood and BM along with increased susceptibility to chemically induced cancers (21). These findings imply that endogenous histamine may facilitate myeloid cell differentiation and cohere with results suggesting that HDC promotes the differentiation of human monocytes into functional antigen-presenting DC (22).

For the present study, we asked if the effects of HDC on the differentiation of myeloid cells may translate into anti-leukemic efficacy. We report that HDC exerts pro-differentiating effects on human monocytic NOX2^+^ AML cells *in vitro* and in immunodeficient mice receiving xenografted human NOX2^+^ AML cells *in vivo*. Our results imply that HDC may exert direct anti-leukemic effects independently of lymphocyte-mediated immunity.

## Materials and Methods

### Monocytic AML Cell Lines

Wild-type (WT) and NOX2-deficient (*NOX2*-KO) clones of the human myelomonoblastic cell line PLB-985 were kindly provided by Dr. Mary Dinauer (Washington University School of Medicine, St. Louis, MO, USA) (23). The OCI-AML3 cell line was obtained from the Tissue Culture Facility at the University of Gothenburg. Cells were maintained at 37°C in 5% CO_2_ in medium containing 10% fetal calf serum, 2 mM l-glutamine, 100 U/ml penicillin, and 100 µg/ml streptomycin. Experiments were performed in medium containing 10% human serum in the presence or absence of HDC (100 µM), 0.5% or 1% dimethyl sulfoxide (DMSO), luminol or isoluminol (100 µg/ml) (Sigma-Aldrich; Munich, Germany). Cell cycle analysis was performed using the BD Pharmingen Bromodeoxyuridine Flow Kit (BD Biosciences; Stockholm, Sweden).

### Analysis of ROS by Chemiluminescence and Fluorescence

Isoluminol-enhanced chemiluminescence was employed to detect superoxide anion production by PLB-985 cells as described (24). Intracellular ROS levels were determined by flow cytometry after incubation according to the manufacturer’s instructions with one of the following fluorescent probes (Thermo Fisher Scientific; Waltham, MA, USA): CellROX Orange (2.5 µM), dihydrorhodamine 123 (10 µM). Mitochondrial ROS were quantified using MitoSOX Red (Thermo Fisher Scientific; 5 µM) with or without antimycin A (Sigma-Aldrich) stimulation.

### Whole-Genome Gene Expression Analysis in HDC-Treated AML Cells

Wild-type and *NOX2*-KO PLB-985 cells were cultured for 48 h with or without HDC (100 µM) or 0.5% DMSO. RNA was isolated using the RNeasy Mini kit (Qiagen; Sollentuna, Sweden) according to the manufacturer’s instructions. Microarray analysis was performed by the Genomics Core Facility at the University of Gothenburg using the HumHT-12 v4 Expression Beadchip (Illumina; San Diego, CA, USA). The microarray gene expression data have been deposited in the Gene Expression Omnibus (accession number GSE100671).

### Generation of Luciferase-Tagged PLB-985 Cells

HEK293T cells (ATCC; Wesel, Germany) were seeded onto a 10 cm plate and transfected with the pHAGE-GFP-luciferase plasmid (#46793) (Addgene; Cambridge, MA, USA) and lentiviral packing plasmids using the standard calcium phosphate precipitation method. The following day medium was discarded and 5 ml fresh medium was added. At 42, 46, 50, and 66 h post-transfection, medium was changed and virus-containing medium was collected, pooled, and passed through a 0.45 µm low protein-binding filter (Sarsted; Nümbrecht, Germany). For cell transduction, 1 ml of filtered medium was added to 4 ml of WT PLB-985 cells (2 × 10^6^ cells/ml). Two passages later the culture was sorted for GFP-positive cells using a three-laser (405, 488, and 633 nm) BD FACSAria II (BD Biosciences).

### Animal Studies

Female *NOD.Cg-Prkdcscid Il2rgtm1Sug/JicTac* (NOG; Taconic Biosciences; Ejby, Denmark) mice were irradiated with 2.5 Gy using the RS-2000 X-ray source (Rad Source Technologies Inc.; Suwanee, GA, USA) after 3 days of receiving antibiotic-supplemented water. On the same day, the mice were engrafted with 2 × 10^6^ WT, *NOX2*-KO or luciferase-tagged PLB-985 variant cells by tail vein injection. HDC (1 mg/mouse) was administered intraperitoneally (i.p.) three times weekly starting 2 weeks after transplantation. Animals showing symptoms of disease were euthanized and BM cells and other tissues were harvested for flow cytometry, including analysis of human CD11b along with human and murine CD45 and histopathology. For the bioluminescence studies, tumor progression was monitored weekly on an IVIS Lumina III XR (Perkin Elmer; Waltham, MA, USA) after i.p. injection of luciferin (150 mg/kg) and anesthesia. All animal experiments were approved by the Research Animal Ethics Committee at the University of Gothenburg.

### Preparation of Primary AML Cells

Peripheral blood samples from 13 newly diagnosed untreated AML patients (Table S1 in Supplementary Material) and buffy coats from healthy donors were obtained from the Sahlgrenska University Hospital, Gothenburg, Sweden. Peripheral blood mononuclear cells (PBMC) were obtained by centrifugation in a Lymphoprep (Axis-Shield; Oslo, Norway) gradient and were then viably frozen. Written informed consent was obtained from all participating patients. The study was approved by the Ethical Committee at the University of Gothenburg.

### Culture and Analysis of Primary Leukemic Cells

The leukemic samples were thawed and CD34^+^ leukemic cells were purified using a Lineage Cell Depletion kit (Miltenyi Biotec; Lund, Sweden) and CD14^+^ monocytic AML cells were isolated using BD IMag CD14 Magnetic Particles (BD Biosciences) according to the manufacturer’s instructions. The purity of the CD34^+^ and CD14^+^ populations was consistently >96 and >98%, respectively, as judged by flow cytometry. The purified cells were cultured in IMDM supplemented with 10% human AB serum and IL-4 (600 U/ml) and GM-CSF (500 U/ml) (both from Peprotech; Stockholm, Sweden) in the presence or absence of HDC at a concentration (100 µM) that optimally saturates histamine H_2_ receptors (H_2_R) (25). After 5 days in culture, cells were analyzed by flow cytometry for expression of maturation markers. Purified CD14^+^ monocytes or CD3^+^ T cells (negative control) from three AML patients with FAB-M4 AML were analyzed for malignant markers using fluorescent *in situ* hybridization or PCR as described in Ref. (11). These analyses verified that the monocytes belonged to the malignant clone. A portion of each sample was refrozen and analyzed at a later time point for H_2_R, NOX2, FPR1, and FPR2 expression by flow cytometry. One patient sample, containing less than 1% viable leukemic cells after the process of refreezing, was excluded from this analysis.

### Flow Cytometry

Flow cytometry was used for phenotype analyses of cultured and xenografted monocytic AML cell lines and primary AML cells. For all flow cytometry analyses, a minimum of 30,000 gated live cells were analyzed on a four-laser BD LSRFortessa (405, 488, 532, and 640 nm; BD Biosciences). Data analysis was performed using FACSDiva software version 8.0.1 (BD Biosciences).

The following anti-human monoclonal antibodies were purchased from BD Biosciences: CD33 (P67.6)-PE-Cy7, CD34 (8G12)-PE, CD11b (ICRF44)-PE, HLA-DR (L243)-APC-Cy7, and CD45 (HI30)-FITC. The secondary antibodies, rat anti-mouse IgG1 (A85-1)-BV421 and rat anti-mouse CD45 (30-F11)-AF700 were also obtained from BD Biosciences. CD14 (TüK4)-Qdot655, goat anti-rabbit IgG-PE-Cy5.5, DAPI, and Live/Dead Fixable Yellow Dead Cell stain were purchased from Thermo Fisher Scientific. Primary anti-H_2_R (LS-A1176) antibody was obtained from LifeSpan Biosciences, Inc. (Seattle, WA, USA) and primary anti-FPR2 (GM1D6) antibody was obtained from Santa Cruz (Heidelberg, Germany). Anti-flavocytochrome b_558_ (NOX2; 7D5)-FITC was purchased from MBL International Corporation (Woburn, MA, USA) and anti-FPR1 (#350418)-APC was from R&D Systems (Minneapolis, MN, USA).

### Reverse Transcription Quantitative PCR (qPCR) Analysis of HDC-Treated AML Cells

Wild-type and *NOX2*-KO PLB-985 cells treated by HDC or DMSO for 2 days were FACS-sorted using a three-laser (405, 488, and 633 nm) BD FACSAria II (BD Biosciences). Triplicates of 100 PLB-985 cells/well were sorted into 96-well plate (Life Technologies) followed by direct cell lysis with each well containing 5 µl of PBS with bovine serum albumin (1 mg/ml; BSA) (26). Samples were then immediately frozen and stored at −80°C until analysis. Reverse transcription was performed at 22°C for 5 min, 42°C for 30 min, and 85°C for 5 min using the TATAA GrandScript cDNA synthesis kit (TATAA Biocenter; Gothenburg, Sweden). qPCR was performed in a CFX384 Touch Real-Time PCR Detection System (Bio-Rad; Solna, Sweden). The thermal cycling profile was 95°C for 1 min, followed by 50 cycles of amplification (95°C for 3 s, 60°C for 30 s, and 72°C for 10 s). Detection of genomic DNA was assessed through inclusion of reverse transcription negative samples in each assay. Cycle of quantification (Cq) values were obtained using the maximum second derivative method. Cq values of samples displaying aberrant amplification curves were removed. qPCR data pre-processing and analysis were performed in GenEx (ver. 6, MultiD). The NormFinder algorithm was used for assessment of seven potential reference genes out of which *EIF1, GAPDH, RPL7*, and *RPS10* were chosen for normalization of gene expression data.

### Statistics

Analysis of the statistical significance of differentially expressed genes between treatment groups was performed using the R software package limma (27). Only genes with a false discovery rate of <0.0001 were considered significant. Heat maps were generated by an unsupervised hierarchical clustering analysis with Euclidian distance matrix using the R function heatmap.2 (R package gplots version 3.0.1). Analysis of microarray data was performed by the Bioinformatics Core Facility at the Sahlgrenska Academy. Group comparisons were performed using two-tailed paired or unpaired *t*-tests. One-way ANOVA followed by Tukey’s multiple comparison test was used for comparisons of three or more groups, and Wilcoxon’s matched-pairs signed rank test was used for comparisons of maturation markers in patient-derived samples. The log-rank test was used to analyze survival curves. *P*-values are designated as follows: *p < 0.05, **p < 0.01, ***p < 0.001, **** *p* < 0.0001. Statistical analyses were performed using GraphPad Prism (San Diego, CA, USA).

## Results

### HDC Promotes Maturation of Monocytic AML Cells by Targeting NOX2

**Figure 1:**
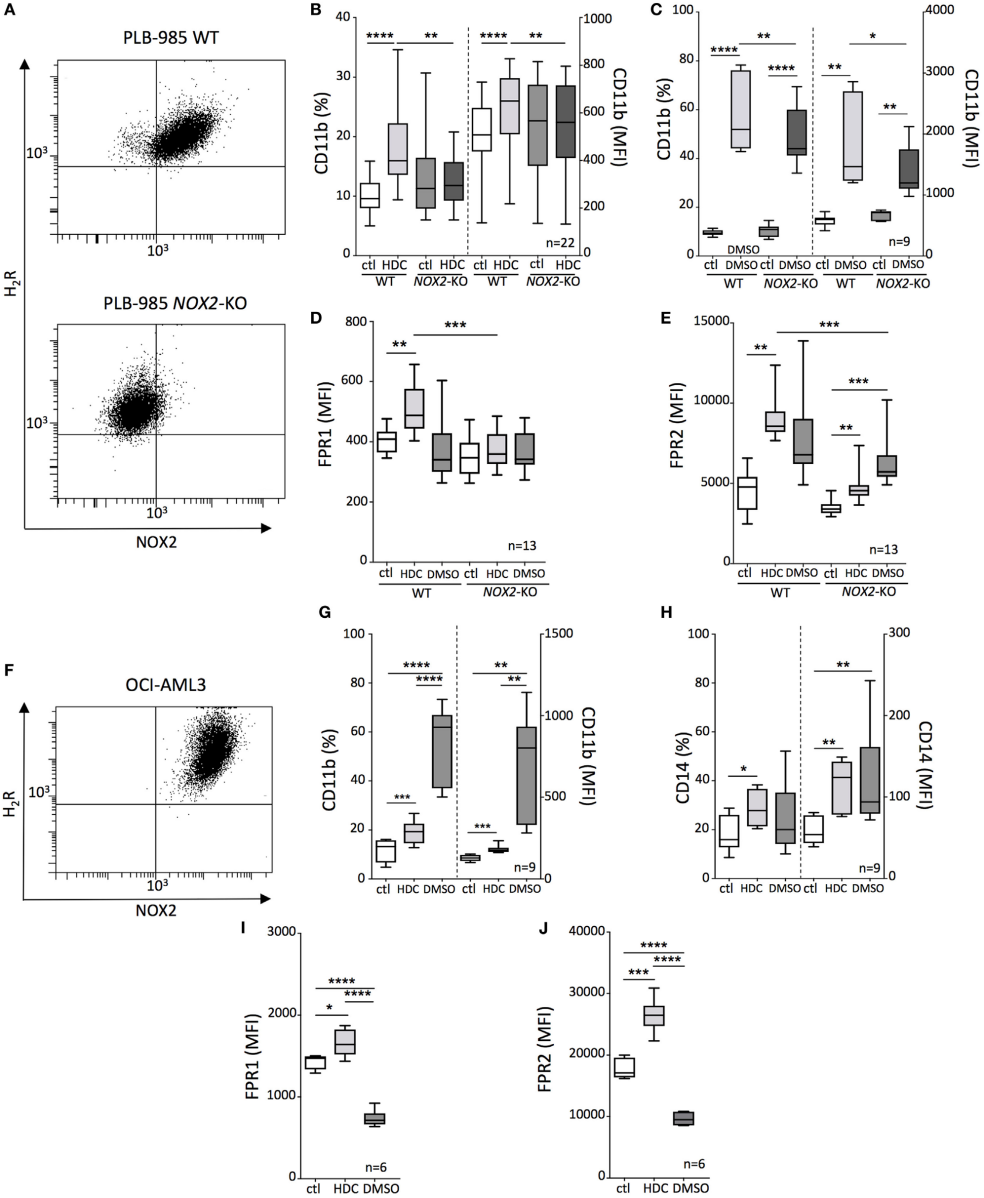
Histamine dihydrochloride (HDC)-induced differentiation of leukemic cells is NOX2-dependent. **(A)** FACS-plots showing NOX2 and H_2_R expression on wild-type (WT) and *NOX2*-KO PLB-985 cells. Expression of CD11b **(B,C)**, FPR1 **(D)**, and FPR2 **(E)** on WT and *NOX2*-KO PLB-985 cells cultured in the presence or absence of HDC or dimethyl sulfoxide (DMSO) as determined by flow cytometry. **(F)** FACS-plot showing NOX2 and H_2_R expression by OCI-AML3 cells. Expression of CD11b **(G)**, CD14 **(H)**, FPR1 **(I)**, and FPR2 **(J)** on OCI-AML3 cells cultured in the presence or absence of HDC or DMSO. Abbreviations: MFI, median fluorescence intensity. ANOVA; *p < 0.05, **p < 0.01, ***p < 0.001, ****p < 0.0001.

The human AML cell lines PLB-985 and OCI-AML3 co-expressed NOX2 and H_2_R (Figures 1A,F) and were employed in the assessment of the pro-differentiating effects of HDC on monocytic AML cells. In addition, the NOX2-deficient counterpart (*NOX2*-KO) of PLB-985 cells was utilized to determine the relevance of NOX2 expression for the actions of HDC. HDC exposure significantly enhanced the expression of CD11b, CD14, formyl peptide receptor-1 and -2 (FPR1 and FPR2) on OCI-AML3 cells, and CD11b, FPR1, and FPR2 on WT PLB-985 cells (Figure 1). The expression of these markers is indicative of a mature monocytic or granulocytic phenotype. HDC did not alter CD11b or FPR1 expression on *NOX2*-KO PLB-985 cells (Figures 1B,D), while FPR2 was also slightly induced by HDC on *NOX2*-KO cells (Figure 1E). The pro-differentiating agent DMSO triggered robust induction of CD11b and CD14 in OCI-AML3 cells (Figures 1G,H) and of CD11b in PLB cells, irrespective of NOX2 expression (Figure 1C). By contrast, DMSO reduced expression of FPR1 and FPR2 in OCI-AML3 cells (Figures 1I,J) and tended to also reduce FPR1 expression in WT PLB-985 cells (Figure 1D).

### Reduced Content of NOX2-Derived and Mitochondrial ROS in NOX2-Deficient AML Cells

**Figure 2:**
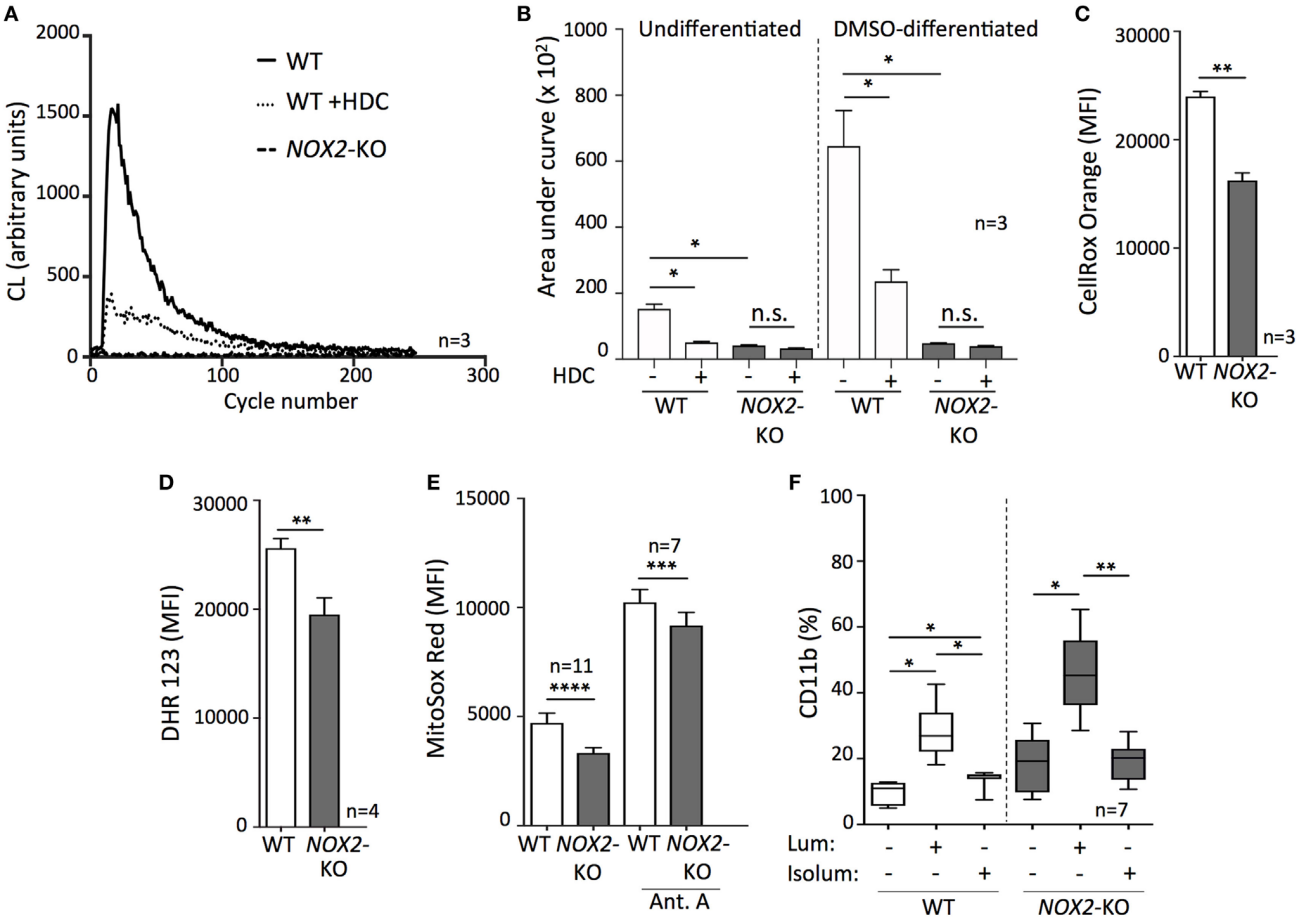
Targeting intracellular reactive oxygen species (ROS) promotes leukemic cell differentiation. **(A,B)** Measurement of ROS production by chemiluminescence following fMLF stimulation in the presence or absence of histamine dihydrochloride. **(A)** A representative curve showing the respiratory burst by dimethyl sulfoxide (DMSO)-differentiated wild-type (WT) or *NOX2*-KO PLB-985. **(B)** ROS production by undifferentiated and DMSO-differentiated WT or *NOX2*-KO PLB-985 cells. **(C–E)** Fluorescent measurement of intracellular ROS in WT and *NOX2*-KO PLB-985 using flow cytometry: **(C)** cytoplasmic ROS, and **(D,E)** mitochondrial ROS. Antimycin A was used to trigger mitochondrial ROS production. **(F)** WT and *NOX2*-KO PLB-985 cells were cultured in the presence or absence of luminol or isoluminol for 5 days and the percentage of CD11b^+^ cells was determined by flow cytometry. Two-tailed paired or unpaired *t*-tests, one-way ANOVA; *p < 0.05, **p < 0.01, ***p < 0.001, ****p < 0.0001.

The only known biological role of NOX2 is to generate ROS (10), and earlier studies show that HDC inhibits NOX2-dependent formation of ROS in human myeloid cells (14, 17, 18, 28). Therefore, we measured NOX2-derived ROS by utilizing a NOX2-activating tripeptide (fMLF) on WT and *NOX2*-KO PLB-985 cells with or without prior DMSO-induced differentiation. These experiments were performed in the presence or absence of HDC. In agreement with an earlier study (22), only NOX2^+^ PLB-985 cells were capable of ROS formation detectable by chemiluminescence, while undifferentiated and DMSO-differentiated *NOX2*-KO PLB-985 did not generate NOX2-derived ROS upon fMLF stimulation (Figures 2A,B). DMSO-differentiated cells generated higher levels of ROS upon stimulation, and HDC significantly prevented fMLF-induced ROS production in undifferentiated as well as in DMSO-differentiated WT AML cells (Figures 2A,B).

Beyond NOX2, ROS are generated by several intracellular sources, including other NOX family members, mitochondria, and peroxisomes (29). To further analyze ROS in WT and *NOX2*-KO PLB-985 cells, we measured the cytoplasmic ROS content from all potential intracellular sources using a fluorescent probe. When compared with WT cells, NOX2-KO cells were found to contain significantly less cytoplasmic ROS (Figure 2C). The reduced ROS levels did not appear to solely depend on the absence of NOX2, as analyses of mitochondrial ROS also showed reduced ROS levels in *NOX2*-KO vs. WT AML cells (Figures 2D,E). In addition to producing less mitochondrial ROS under resting conditions, the ROS-producing capacity of *NOX2*-KO cells remained significantly diminished upon stimulation with antimycin A (Figure 2E), a disruptor of mitochondrial electron transport (30).

To further elucidate the effects of ROS inhibition on leukemic cell maturation we utilized luminol and isoluminol, both of which neutralize ROS by scavenging superoxide anion and hydrogen peroxide (31). The membrane-permeable luminol robustly upregulated CD11b in WT and *NOX2*-KO PLB-985 cells. In contrast, isoluminol, which is membrane-impermeable and thus only scavenges extracellular ROS (32, 33), was less efficacious in triggering monocytic AML cell maturation, and did so only in WT *NOX2*^+/+^ PLB-985 cells (Figure 2F).

### HDC Modulates Target Gene Expression in NOX2-Expressing AML Cells

**Figure 3:**
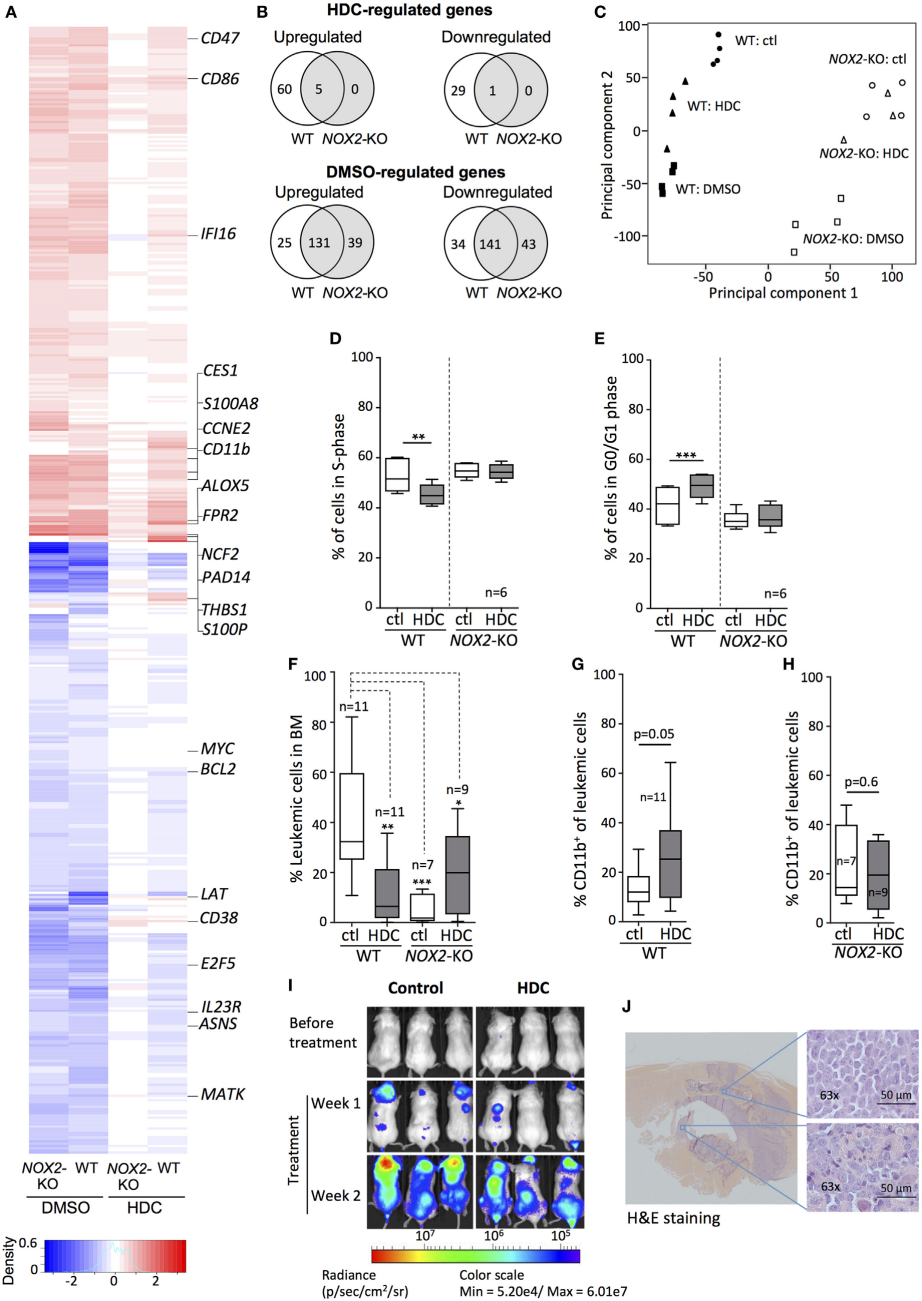
Histamine dihydrochloride (HDC) selectively regulates gene expression in NOX2^+^ leukemic cells and exerts anti-leukemic efficacy *in vivo*. **(A–C)** Wild-type (WT) and NOX2-KO PLB-985 cells were cultured in the presence or absence of HDC or dimethyl sulfoxide (DMSO) for 48 h. RNA was extracted and microarray analysis performed. **(A)** Heat map showing genes significantly (*p* < 0.0001) differentially expressed in response to at least one stimulus. Data were calculated as gene expression after stimulation divided by gene expression in the matching unstimulated control sample (*n* = 4). The color intensity represents the ratio of expression of treated vs. control for the indicated cell type. Upregulated genes: red; downregulated genes: blue. **(B)** Venn diagrams of genes from the heat map that were significantly (*p* < 0.05) up- or downregulated by HDC or DMSO in WT and *NOX2*-KO cells. **(C)** Principal component analysis based on microarray genes for control- (ctl), HDC-, and DMSO-treated for WT and *NOX2*-KO PLB-985 cells displaying clustering based on the first two principal components. Percentage of cells in **(D)** S-phase and **(E)** G0/G1 phase for WT and *NOX2*-KO PLB-985 cells cultured with or without HDC for 5 days. **(F–J)** WT and *NOX2*-KO PLB-985 cells were engrafted into NOD/SCID-IL2RG (NOG) mice followed by systemic HDC treatment. **(F)** Percentage of leukemic cells (CD45^+^ human cells) in bone marrow (BM) and **(G,H)** percentage CD11b^+^ leukemic cells of total human CD45^+^ in BM at death. **(I)** Bioluminescence from luciferase-tagged WT PLB-985 cells injected into NOG mice before and after treatment with HDC or control (saline). **(J)** Hematoxylin and eosin stained spinal section from a *NOX2*-KO PLB-985 injected mouse with hindlimb paralysis. Extracted images show leukemic cell infiltration into BM and spinal ganglion. Overview image was produced by stitching of 10× tiles. Images were obtained using the AxioObserver/Apotome (Zeiss; Oberkochen, Germany). Paired *t*-test, one-way ANOVA; *p < 0.05, **p < 0.01, ***p < 0.001.

Next, we examined HDC-induced gene expression changes by culturing WT and *NOX2*-KO PLB-985 cells with or without HDC followed by microarray analysis of whole-genome gene expression. DMSO, a non-specific inducer of cellular differentiation (34, 35), was used for comparison. As shown in Figures 3A,B, HDC modulated the expression of a subset of genes in WT AML cells, whereas virtually no genes were significantly affected by HDC in *NOX2*-KO cells. By contrast, WT and *NOX2*-KO cells displayed comparable gene expression in response to DMSO (Figures 3A,B). Principal component analysis (PCA), employed to discern patterns in the microarray data, revealed that WT cells were organized into three distinct clusters: control-, HDC-, and DMSO-treated cells (Figure 3C). *NOX2*-KO cells formed two clusters in which control- and HDC-treated cells grouped together, whereas cells treated with DMSO formed a separate cluster (Figure 3C).

Sixteen genes were uniquely modulated by HDC in WT cells (Figure S1 in Supplementary Material), among them the early differentiation marker *CD38* and *S100P*, a regulator of cell cycle progression and differentiation (36, 37). HDC as well as DMSO triggered enhanced transcription of maturation markers, such as *CD11b, CD86, FPR2*, and the NOX2 complex component p67^phox^ (*NCF2*) along with modulated expression of cell cycle regulators, such as *ASNS, E2F5, CCNE2*, and *THBS1* (Figure 3A). We confirmed the validity of the microarray expression data by RT-qPCR analyses of 23 genes with documented roles in differentiation, proliferation, ROS production, apoptosis, and immune function (Figure S2 in Supplementary Material). A lower degree of correlation was observed for HDC-treated *NOX2*-KO cells likely due to the low gene expression induced under these conditions.

### HDC Blocks S-Phase Entry in Leukemic Cells

The microarray and RT-qPCR data implied that gene expression relevant to cell cycle progression was specifically regulated by HDC in WT but not in *NOX2*-KO AML cells. We, therefore, monitored the cell cycle progression of HDC-treated WT and *NOX2*-KO PLB-985 cells. HDC exposure decreased the fraction of WT cells entering S-phase (Figure 3D), while a larger fraction of cells remained in G0/G1 phase (Figure 3E). *NOX2*-KO PLB-985 cells showed no sensitivity to HDC-induced cell cycle modulation (Figures 3D,E). Similar effects of HDC on cell cycle progression were observed in OCI-AML3 cells (Figure S3 in Supplementary Material).

### HDC Reduces the Burden of Monocytic Leukemia and Promotes Leukemic Cell Maturation *In Vivo*

To determine the effects of HDC on the expansion and maturation of leukemic cells *in vivo*, we utilized an AML xenograft model in which WT and *NOX2*-KO PLB-985 cells were transplanted into immunodeficient NOG mice. The transplanted NOG mice developed leukemia after engraftment of AML cells with a latency of 30–50 days. At the time of sacrifice the BM of HDC-treated mice transplanted with WT AML cells contained significantly fewer CD45^+^ leukemic cells, compared to control-treated mice (32 ± 21 vs. 7 ± 13 of all hematopoietic cells, median ± SD). In contrast, the frequency of *NOX2*-KO AML cells in BM was unaffected by HDC treatment, and was significantly lower than the frequency of leukemic cells in BM of mice transplanted with WT AML cells (Figure 3F). Further characterization of the engrafted WT AML cells showed that HDC treatment tended to increase cell surface expression of CD11b (Figure 3G), which was not observed for engrafted *NOX2*-KO cells (Figure 3H). Experiments using NOG mice engrafted with bioluminescent luciferase-tagged WT PLB-985 cells supported that *in vivo* HDC treatment reduced leukemic cell expansion (Figure 3I).

Histamine dihydrochloride treatment also slightly but significantly increased the overall survival of WT (*p* = 0.04, log-rank test), but not of *NOX2*-KO PLB-985-engrafted mice (*p* = 0.8, log-rank test) (Figure S4 in Supplementary Material). In this model, the majority of engrafted mice developed extramedullary myeloid sarcoma (38) consisting of CD45^+^ human leukemic cells. The sarcomas infiltrated the spinal column (Figure 3J), which commonly resulted in hind limb paralysis after which the mice were euthanized. Myeloid sarcomas were apparent in both WT and *NOX2*-KO PLB-985 cell-engrafted mice and were seemingly not significantly impacted by *in vivo* treatment with HDC.

### HDC Promotes the Maturation of Primary Monocytic AML Cells

**Figure 4:**
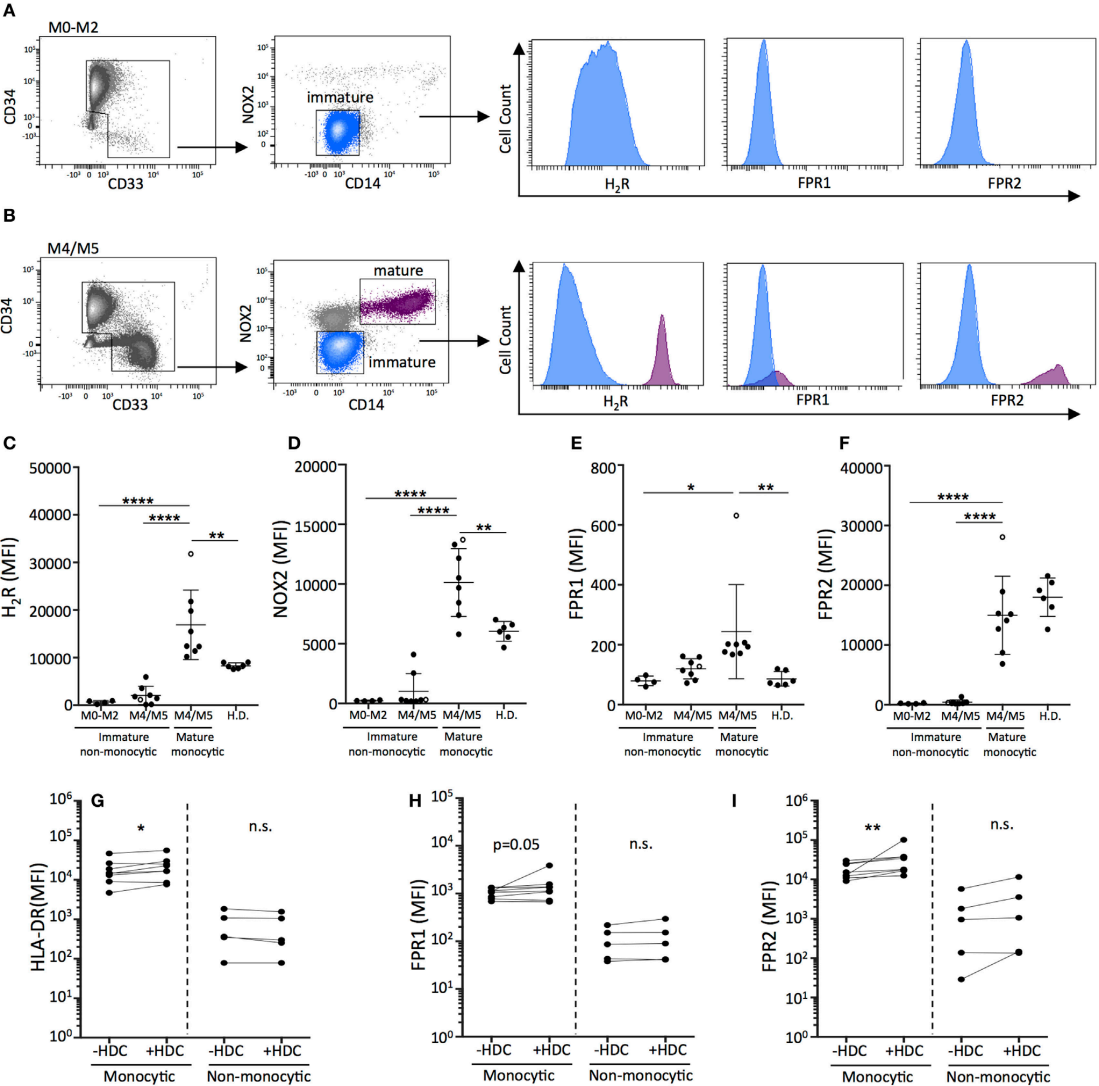
Histamine dihydrochloride (HDC) facilitates the differentiation of monocytic primary leukemic cells and may be preferentially efficacious in monocytic forms of leukemia. FACS-plots showing live peripheral blood mononuclear cells from representative newly diagnosed patients with **(A)** FAB-M0 acute myeloid leukemia (AML) with a dominant immature leukemic population (CD34^+^CD33^−^CD14^−^) and **(B)** FAB-M4 AML with two distinct populations: an immature blast population (CD34^+^CD33^−^CD14^−^) and a mature monocytic population (CD34^−^CD33^+^CD14^+^). The expression of **(C)** H_2_R, **(D)** NOX2, **(E)** FPR1, and **(F)** FPR2 on primary AML cells [gated as indicated in **(A,B)**] and monocytes from healthy donors was determined by flow cytometry. The M5 leukemia is represented by an open circle. One-way ANOVA. **(G–I)** Median fluorescence intensity as determined by flow cytometry of **(G)** HLA-DR, **(H)** FPR1, and **(I)** FPR2 on live primary monocytic AML cells (FAB: M4/M5) or non-monocytic AML cells (FAB: M0–M2) cultured for 5 days with GM-CSF/IL-4 in the presence or absence of HDC. Wilcoxon matched pair’s test. *p < 0.05, **p < 0.01, ***p < 0.001, ****p < 0.0001.

We next investigated the differentiation of primary leukemic cells from AML patients in response to HDC. For these studies, NOX2 and H_2_R expression on AML cells was determined in newly diagnosed patients (Table S1 in Supplementary Material). In agreement with previous studies (11), mature monocytic AML cells of FAB-classes M4 and M5 co-expressed NOX2 and H_2_R, while leukemic cells recovered from patients with non-monocytic AML (FAB-M0, M1, and M2) cells did not (Figures 4A–D). Moreover, mature monocytic AML cells expressed significantly higher levels of FPR1 and FPR2, which are additional markers of maturation and mediators of phagocyte chemotaxis and NOX2 activation (39, 40), compared with non-monocytic AML cells (Figures 4E,F). Mature malignant monocytes expressed higher levels of H_2_R, NOX2, and FPR1 compared with monocytes recovered from healthy controls (Figures 4C–E). The single M5 leukemia included showed the highest expression levels for all maturation markers on mature monocytic cells. Exclusion of this data point did not reduce the statistical significance between groups. The healthy controls were likely younger than the patients in this study. However, when patient samples were dichotomized based on age no significant differences were observed in H_2_R, NOX2, FPR1, and FPR2 expression between younger (<60) and older (>60) patients (Figure S5 in Supplementary Material).

In further experiments, primary human AML cells were cultured in the presence or absence of HDC followed by analysis of expression of the maturation markers HLA-DR, FPR1, and FPR2. In samples from patients with monocytic AML (FAB-M4/M5), exposure to HDC significantly enhanced the expression of the antigen presentation marker HLA-DR (Figure 4G) and triggered expression of FPR1 (Figure 4H) and FPR2 (Figure 4I) on the leukemic CD14^+^ monocytes. Analysis of sorted leukemic cells confirmed that the isolated monocytic CD14^+^ cells belonged to the leukemic clone (Table S1 in Supplementary Material). Leukemic CD34^+^ cells recovered from patients of FAB-M0, M1, or M2 did not show signs of maturation upon HDC exposure (Figures 4G–I).

## Discussion

Although several immunotherapeutic regimens are currently evaluated for relapse prevention in AML (41–45), only the combination of HDC/IL-2 has yielded positive results over standard-of-care in a randomized setting (4). Thus far, HDC-based therapy has been evaluated only in patients in CR, when the tumor burden is low. In the present study, we show that HDC exerts direct effects on leukemic cell maturation and cell cycle progression *in vitro* that may translate into anti-leukemic efficacy *in vivo*, even in the absence of lymphocyte-mediated immunity. HDC thus promoted NOX2-dependent maturation and differentiation of human leukemic cell lines of monocytic origin and exerted similar actions on primary leukemic cells of myelomonocytic and monocytic origin. Additionally, HDC modulated the expression of cell cycle-related genes, including *CCNE2, E2F5*, and *ASNS*, and blocked the progression of monocytic leukemic cells through the cell cycle.

The HDC-induced maturation and inhibition of proliferation were dependent on the presence of functional NOX2. In agreement with these *in vitro* findings, the administration of HDC to immunodeficient mice reduced the expansion of xenografted NOX2-sufficient PLB-985 cells, but not of *NOX2*-KO PLB-985 cells. Characterization of the redox status of PLB-985 cells revealed that, in addition to the inability of *NOX2*-KO cells to produce NOX2-derived ROS, mitochondrial ROS production was reduced in these cells compared with WT cells. These findings agree with previous studies demonstrating crosstalk between intracellular ROS-generating sources (46, 47).

In the studies of leukemic cell differentiation, we sought to define the specific role of intra- and extracellular ROS by using the ROS scavengers, luminol and isoluminol (31, 32). Luminol exposure yielded a more robust differentiation phenotype than isoluminol implying that intracellular ROS in leukemic cells impede maturation. Luminol promoted the differentiation of WT as well as of *NOX2*-KO leukemic cells implying that non-NOX2 ROS sources, including ROS generated by other NOX family members or during mitochondrial respiration, may have contributed to the observed deficiency of leukemic cell maturation. Interestingly, despite that *NOX2*-KO leukemic cells exhibited significantly reduced levels of mitochondrial and cytoplasmic ROS compared with WT cells, luminol induced a pronounced differentiation of these cells, presumably by scavenging a remaining pool of intracellular ROS. Primary human AML cells reportedly contain a higher mass of mitochondria, compared with nonmalignant hematopoietic cells (13, 48, 49), although this may not translate into elevated amounts of mitochondrial ROS (47). Further studies are warranted to define the potential role of mitochondria as well as NOX1 and NOX4, both of which may be expressed by primary leukemic cells in addition to NOX2 (47), for the maturation and expansion of myeloid leukemic cells.

The *in vivo* study largely mimicked the *in vitro* results in that HDC administration to immunodeficient NOG mice reduced the expansion of xenografted monocytic leukemic cells in BM and also tended to induce a more mature phenotype (CD11b^+^ cells) within the leukemic cell population. These effects of HDC were absent in mice engrafted with *NOX2*-KO monocytic AML cells. HDC treatment also slightly prolonged the survival of NOG mice engrafted with NOX2-sufficient AML cells, but did not impact on the survival of mice carrying *NOX2*-KO xenografts. These findings imply that pharmacological inhibition of NOX2 is a conceivable anti-leukemic strategy in monocytic AML. The notion that functional NOX2 may promote the expansion of NOX2^+^ leukemia is supported by results showing that *NOX2* knockdown in OCI-AML3 cells prolongs the survival of xenografted mice in an AML model (13) and that genetic ablation of *NOX2* from hematopoietic cells reduces the *in vivo* expansion of murine *BCR-ABL1*^+^ leukemic cells in a CML model (50).

Notably, the development of extramedullar myeloid leukemia was significantly rate-limiting for the survival of mice receiving PLB-985 xenografts. However, the HDC-treated mice showed significantly reduced levels of leukemic cells in BM, and in addition, mice engrafted with *NOX2*-KO leukemic cells showed lower frequency of leukemic cells in BM compared with mice xenoengrafted with WT leukemic cells when sacrificed due to the development of myeloid sarcoma. Similar results were reported by Ågerstam et al. (38), where NOD/SCID mice engrafted with human AML cells showed reduced frequency of leukemic cells in BM after treatment with an antibody against a stem cell antigen (anti-IL1RAP) but also developed extensive myeloid sarcoma that was rate-limiting for survival. The possibility that myeloid sarcoma and extramedullary leukemia may be controlled by immune effector mechanisms that are absent in NOG or NOD/SCID mice should be further investigated.

We conclude that HDC, by targeting NOX2 expressed by monocytic leukemic cells, exerts anti-leukemic efficacy *in vitro* and *in vivo* also in the absence of cytotoxic effector lymphocytes. These properties of HDC may be relevant to the proposed clinical benefit of HDC-based therapy in monocytic forms of AML and merit the study of additional strategies to target NOX2-derived ROS in NOX2^+^ myeloid leukemias.

## Ethics Statement

This study was carried out in accordance with the recommendations of guideline 86-2014, Research Animal Ethics Committee at the University of Gothenburg. The protocol was approved by the Research Animal Ethics Committee at the University of Gothenburg. The study on human primary leukemia cells was carried out in accordance with ethics 228-12, approved by the ethical committee at Gothenburg University.

## Author Contributions

RK designed the research, performed experiments, analyzed data, and wrote the manuscript. EA and BL performed experiments. HGW and MSN performed experiments and data analysis. JA performed data analysis. JAN and AS provided technical expertise and access to equipment. FBT contributed critical input and edited the manuscript. KH provided advice and wrote the manuscript. AM conceived and supervised the project, designed the research, and wrote the manuscript. The manuscript was prepared with input from all authors.

## Conflict of Interest Statement

HGW, FBT, KH, and AM are authors of issued or pending patents protecting the use of HDC in cancer immunotherapy.
