# Supplementary material for: Anti-Leukemic Properties of Histamine in Monocytic Leukemia: The Role of NOX2
Source: Front Oncol. 2018 Jun 18;8:218. doi: 10.3389/fonc.2018.00218 (PMC6015904; doi:10.3389/fonc.2018.00218)
Supplement: Supplementary file 1 [file Data_Sheet_1.PDF]

Supplementary Material

Anti-leukemic properties of histamine in monocytic leukemia: the role of NOX2

Roberta Kiffin[1], Hanna Grauers Wiktorin[1], Malin S. Nilsson[1], Johan Aurelius, Ebru Aydin[1], Brianna Lenox[1], Jonas A. Nilsson[1], Anders Ståhlberg[1], Fredrik Bergh Thorén, Kristoffer Hellstrand[1] and Anna Martner[1*]

[1]Sahlgrenska Cancer Center, University of Gothenburg, Gothenburg, Sweden

*** Correspondence:**
Anna Martner
anna.martner@gu.se

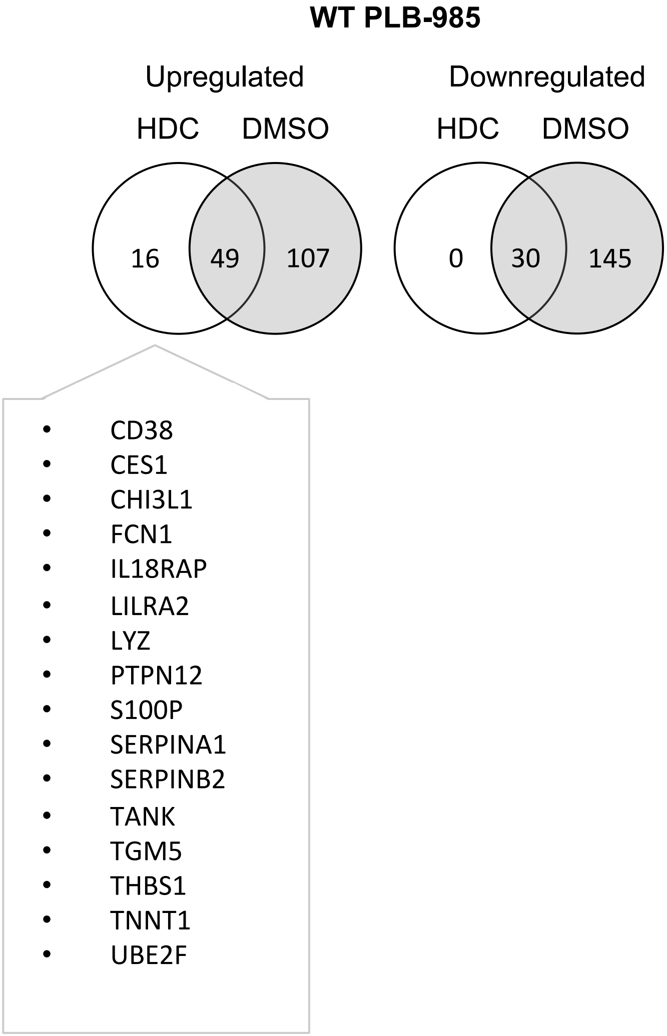

Supplementary Figure 1. HDC regulates a limited subset of genes compared to DMSO. Venn diagrams of genes upregulated and downregulated by HDC and DMSO in WT PLB-985 cells relative to control. Only heatmap genes with a p-value <0.05 and log FC>0.5 for upregulated genes and log FC<0.5 for downregulated genes were included in the Venn diagrams.

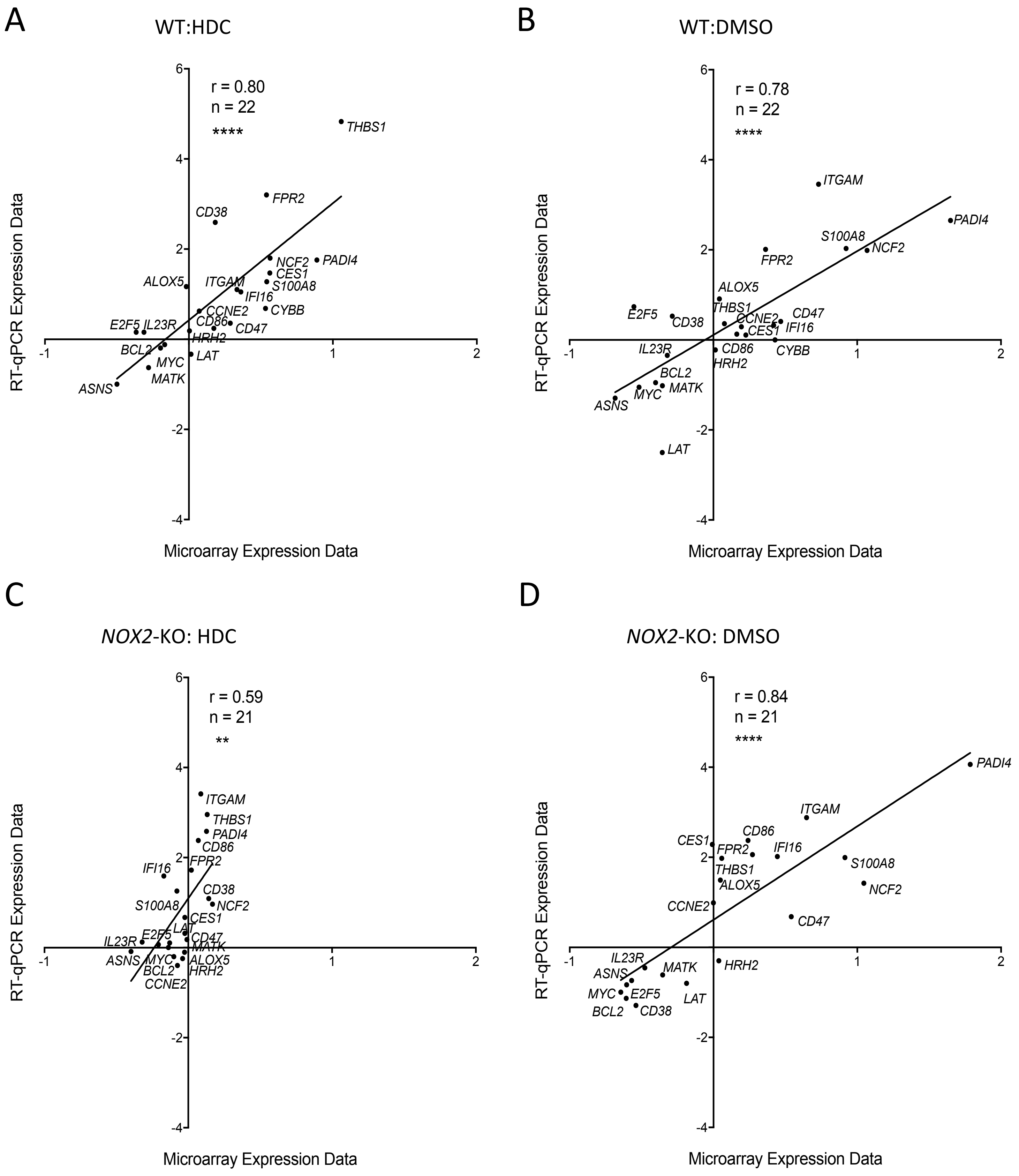

Supplementary Figure 2. Confirmation of microarray results with reverse transcription quantitative PCR. To verify the microarray results, three replicates of one hundred cells were FACS-sorted from control-, HDC- or DMSO-treated wild-type (WT) or *NOX2*-KO (*NOX2*-KO) PLB-985 cells. The cell lysates were then subjected to RT-qPCR quantification of expression of 21 genes of interest. The graphs illustrate the correlations (Pearson's *r*) between the RT-qPCR and microarray expression data, displayed as $\log_2$ fold change from control in **(A)** HDC-treated WT cells, **(B)** DMSO-treated WT cells, **(C)** HDC-treated KO cells, and **(D)** DMSO-treated KO cells. **p<0.01, ****p<0.0001.

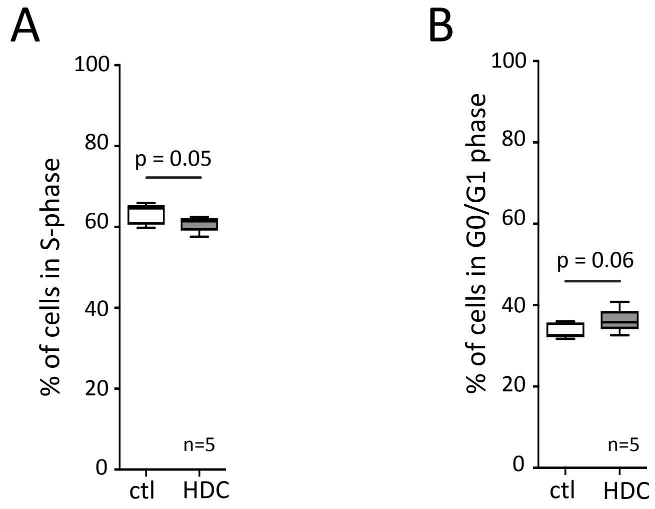

Supplementary Figure 3. HDC inhibits S-phase entry. OCI-AML3 cells were cultured for five days in the presence or absence of HDC. Percentage of cells in S-phase **(A)** and G0/G1 **(B)** phase was determined by BrdU incorporation and flow cytometry. Paired t test.

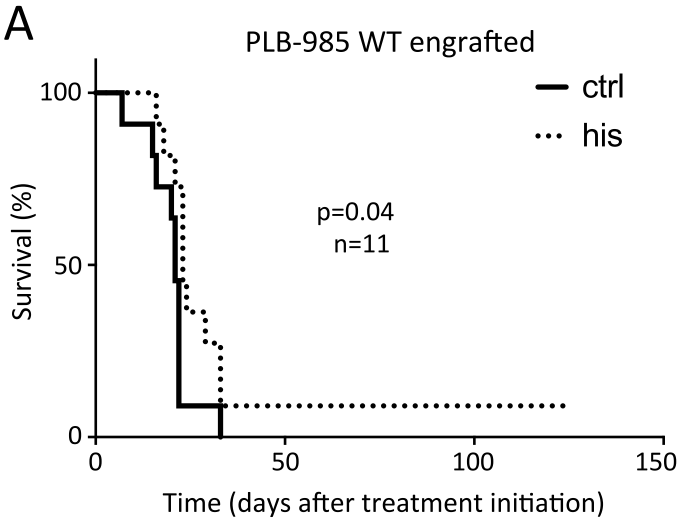

Supplementary Figure 4. Overall survival in AML engrafted mice. Kaplan-Meier plots showing survival of control and HDC-treated (**A**) WT and **(B)** *NOX2*-KO PLB-985 engrafted NOG mice. Log-rank test. *p<0.05.

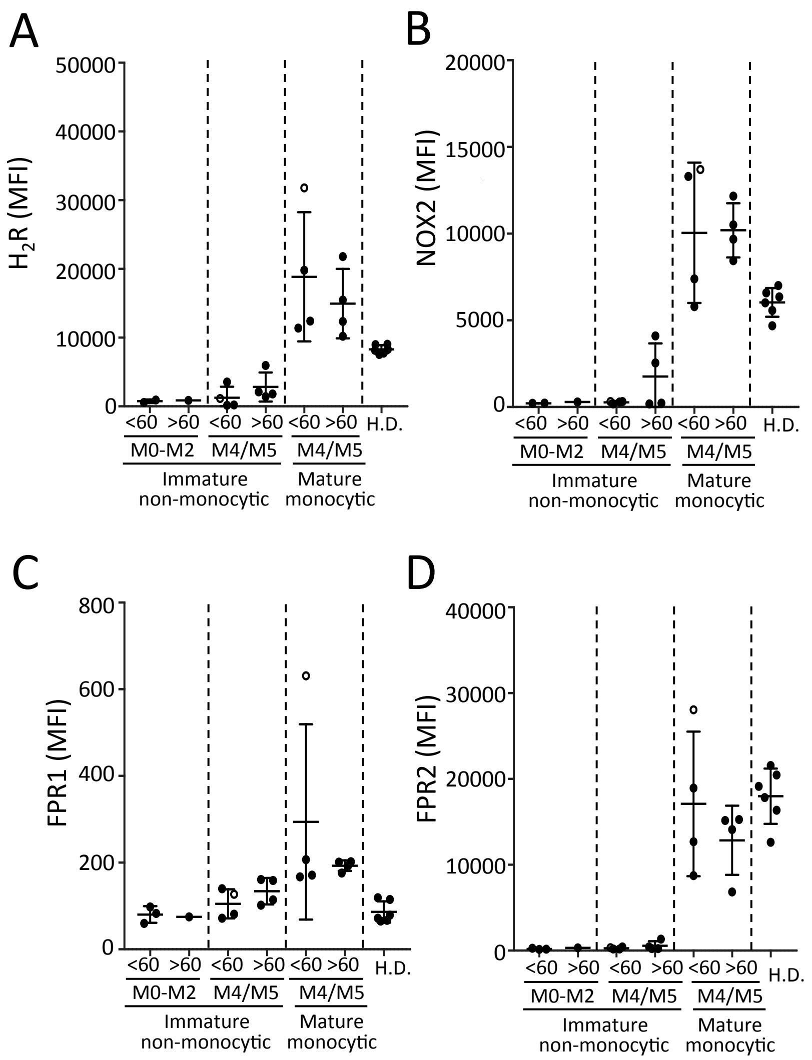

Supplementary Figure 5. Age independent expression of markers. Patient samples were divided based on age into 2 groups (<60 and >60) and expression (median fluorescent intensity, MFI) of **(A)** H_2R **(B)** NOX2, **(C)** FPR1, and **(D)** FPR2 were analyzed. The expression by healthy donor monocytes is shown as a comparison. Unpaired t test.

Supplementary Table 1. Characterization of newly diagnosed AML patients. n.a. –not available

**Patient**	**FAB class**	**Age**	**Sex**	**Cytogenetics**	**Molecular genetics**
1	M4	73	n.a.	n.a.	n.a.
2	M4	77	M	Normal Karyotype	FLT3-ITD^{+}
3	M4	48	F	Normal Karyotype	NPM1^{+}
4	M5	33	n.a.	n.a.	n.a.
5	M4	66	n.a.	n.a.	n.a.
6	M4	77	n.a.	n.a.	n.a.
7	M4	39	n.a.	n.a.	n.a.
8	M4	23	M	t(16;16), -21q	None detected
9	M1	71	n.a.	n.a.	n.a.
10	M0	42	n.a.	n.a.	n.a.
11	M1	57	n.a.	n.a.	n.a.
12	M2	59	n.a.	n.a.	n.a.
13	M0	n.a.	n.a.	n.a.	n.a.
